# Grape-Derived Exosome-like Nanoparticles Ameliorate Prediabetes in Mice via Amino Acid Metabolic Reprogramming

**DOI:** 10.3390/ijms27156947

**Published:** 2026-08-02

**Authors:** Zaoling Liu, Shuya Cai, Aerna Qiayimaerdan, Yile Tang, Jin Su, Zisen Yang

**Affiliations:** Department of Epidemiology and Health Statistics, School of Public Health, Xinjiang Medical University, Urumqi 830011, China

**Keywords:** targeted metabolomics, untargeted metabolomics, diabetes, grape exosomes

## Abstract

This study investigates the effects of grape-derived exosomes on prediabetic mice and elucidates their underlying mechanisms through an integrated metabolomics approach. A prediabetic mouse model was established, with 18 mice randomly assigned to three groups: model control group, grape exosome treatment group, and antioxidant nutrient supplementation group. Therapeutic efficacy was assessed using fasting plasma glucose (FPG) and homeostasis model assessment of β-cell function (HOMA-β). Intestinal content metabolites were profiled via both untargeted ultra-high performance liquid chromatography coupled with orbitrap electrospray mass spectrometry (UHPLC-OE-MS) and targeted metabolomics analyses. Grape exosomes effectively mitigated prediabetes progression. Untargeted metabolomics revealed 1222 downregulated and 425 upregulated metabolites in the grape exosome group relative to the model control group, among which riboflavin (upregulated) was potentially associated with amino acid metabolism. Targeted metabolomics identified 23 upregulated and 41 downregulated metabolites, with glycine showing a significant elevation. Notably, both the grape exosome group and antioxidant nutrient supplementation group exhibited increased glycine levels and shared enriched metabolic pathways, including D-glutamine and D-glutamate metabolism, phenylalanine, tyrosine and tryptophan biosynthesis, as well as valine, leucine, and isoleucine biosynthesis. Integrated untargeted and targeted metabolomics analysis demonstrates that grape exosomes ameliorate diabetes-related metabolic disorders and slow the progression of prediabetes, indicating that grape-derived exosomes have great potential as a functional intervention agent for early glucose metabolism dysfunction.

## 1. Introduction

Diabetes mellitus (DM) is a multifaceted metabolic disorder driven by genetic and environmental interactions [1,2]. With global prevalence rising steadily [3,4], projections estimate 578 million adults will be affected by 2030, increasing to 693 million by 2045 [5]. Type 2 DM (T2DM) elevates risks for cardiovascular complications and diminishes quality of life [6]. Notably, the greatest relative increase in prevalence is projected in middle-income countries, with global health expenditures expected to exceed 1 trillion USD by 2045 [7]. In parallel, China has seen a 56% surge in T2DM cases over the past decade (90 to 140 million) [6]. Given the urgent need for cost-effective prevention, particularly targeting prediabetes—a reversible precursor to T2DM [8]—targeted interventions are paramount.

Pharmacological management integrates Western medicine and Traditional Chinese Medicine (TCM), with plant-based functional foods offering distinct advantages for chronic use. Beyond conventional phytochemicals [9], plant-derived microRNAs (miRNAs) have emerged as a novel cross-kingdom regulatory axis. Seminal work by Zhang et al. established that exogenous plant MIR168a survives digestion, enters circulation, and regulates mammalian gene expression [10]. Subsequent studies extended this paradigm to edible plant nanoparticles, which modulate inflammation and metabolic disorders across species boundaries [11]. These bioactive RNAs mediate the health benefits of plant-based diets via cross-kingdom gene regulation [12].

Xinjiang’s distinct geography yields a wealth of medicinal flora, notably Vitis vinifera. Beyond ethnopharmacology, grape-derived exosome-like nanoparticles (GELNs) have attracted international attention as edible nanoparticles capable of crossing the intestinal mucus barrier and delivering bioactive cargos [13]. Indigenous cultivars (e.g., Manai and SuoSuo) have served as Uyghur medicinal staples for centuries, supporting hepatobiliary function [14]. Beyond their rich profile of antioxidants and bioactive metabolites [15], grape-derived compounds are known to ameliorate hyperglycemia and dyslipidemia, restore pancreatic islet β-cell function, and modulate cellular proliferation and apoptosis [16]. However, whether grape-derived miRNAs encapsulated in GELNs contribute to these effects via cross-kingdom regulation remains unknown.

Building on the established role of plant-derived exosomes in cross-kingdom communication and the ethnopharmacological significance of grapes in Uyghur medicine, we hypothesized that grape-derived exosome-like nanoparticles (GELNs) mitigate prediabetes by orchestrating specific metabolic pathways.

Metabolomics provides a high-throughput window into the endogenous metabolite landscape of biofluids and tissues. While untargeted approaches offer an unbiased snapshot of global metabolic profiles, targeted analyses quantify specific pathways of interest. Integrating these strategies facilitates robust biomarker discovery and mechanistic elucidation of therapeutic interventions [17].

Applying this integrated workflow to nutritional interventions, we established a prediabetic mouse model and orally administered grape-derived exosome-like nanoparticles (GELNs) supplemented with vitamin C, vitamin E, and selenium. Our prior work established that these antioxidant nutrients preserve cellular function and mitigate oxidative damage while exhibiting hypoglycemic potential [18]; we recently demonstrated that their antioxidant activity directly attenuates diabetes onset [19]. Using an integrated untargeted and targeted metabolomics approach over an 8-week intervention, we evaluated the therapeutic efficacy of GELNs and elucidated the mechanisms by which grape exosomes modulate prediabetes, thereby providing a mechanistic foundation for novel preventive and therapeutic strategies.

## 2. Results

### 2.1. Grape-Derived Exosomes Can Ameliorate the Condition of Mice with Prediabetes

**Figure 1:**
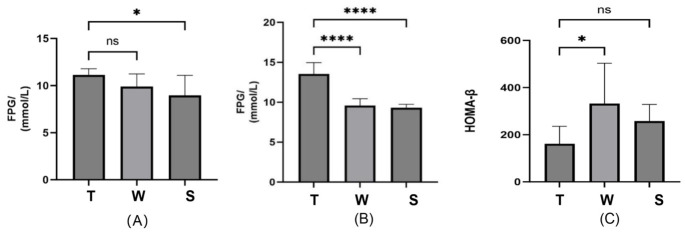
The impact of interventions on mice with prediabetes. (**A**) Fasting blood glucose levels in mice after 4 weeks of gavage; (**B**) Fasting blood glucose levels in mice after 8 weeks of gavage; (**C**) Insulin beta-cell index in mice across groups. **** indicates *p* < 0.0001, * indicates *p* < 0.05, and ns indicates *p* > 0.05. T: Model control group, W: Grape exosome group, S: Antioxidant nutrient group.

The results demonstrated that mice receiving grape exosome-like nanoparticles (GELNs; W group) or antioxidant nutrients (S group) showed significantly lower fasting plasma glucose (FPG) levels than the model control group (T group) after both 4 and 8 weeks of oral gavage (Figure 1A,B). Consistently, homeostatic model assessment of β-cell function (HOMA-β) levels were elevated in these intervention groups compared with the T group (Figure 1C). Collectively, these findings indicate that GELN and antioxidant nutrient treatments effectively delay the progression of prediabetes—highlighting their potential as interventions for early-stage glucose dysregulation.

### 2.2. The Effects of the Intervention Measures on FINS, HOMA-IR, and HOMA-β in the Diabetic Preclinical Model Mice

Following an eight-week intragastric administration regimen, we assessed the impact of interventions on systemic insulin dynamics in preclinical diabetic mice (Table 1). Compared with the model control group, fasting insulin (FINS) levels were significantly reduced in both the GELN-treated (2.37 ± 0.17 ng/mL) and antioxidant nutrient (2.22 ± 0.09 ng/mL) groups (both *p*< 0.05). Intergroup comparisons further revealed significant heterogeneity in the homeostasis model assessment of insulin resistance (HOMA-IR) (*p*< 0.05). In contrast, the HOMA-β index, reflecting β-cell function, remained statistically comparable across all experimental groups (*p*> 0.05).

### 2.3. The Results of Untargeted Metabolomics

#### 2.3.1. Metabolic Principal Component Analysis (PCA)

**Figure 2:**
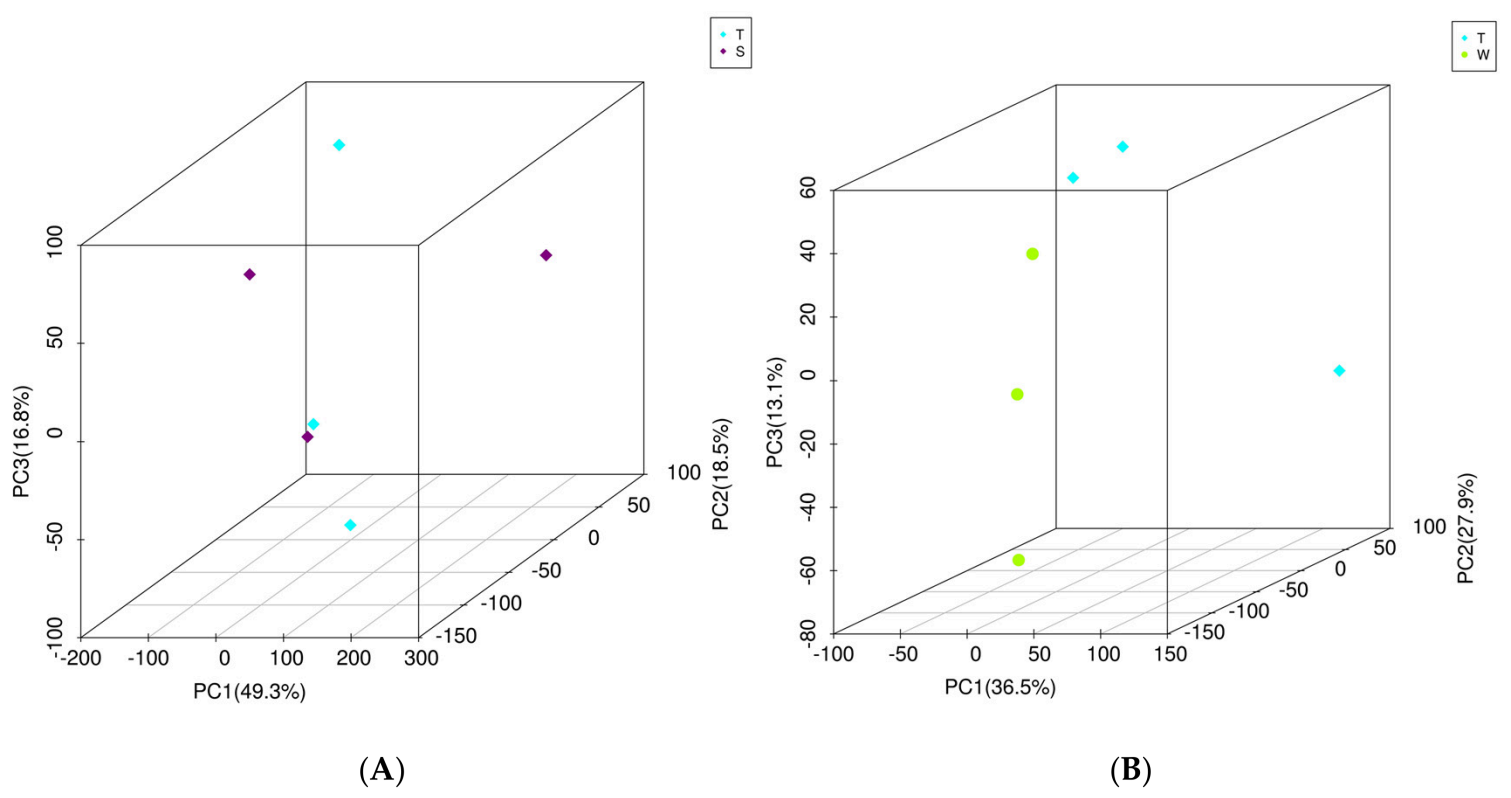
Three-dimensional scatter plot of the PCA model. (**A**) The comparison between the model control group and the antioxidant nutrient group; (**B**) The comparison between the model control group and the grape exosome group.

Principal Component Analysis (PCA) is an unsupervised statistical technique that uses orthogonal transformation to convert a set of potentially correlated observed variables into linearly uncorrelated principal components. As an unsupervised learning method, PCA uncovers the intrinsic structure of metabolomics data, enabling a more intuitive interpretation of variable relationships. It effectively visualizes two key aspects: the overall distribution patterns of metabolic profiles and the inter-group variability between sample sets (Figure 2). Importantly, sample proximity in PCA plots reflects metabolic similarity—closer samples share more similar metabolite types and concentrations, while distant samples indicate larger differences in overall metabolic phenotypes. In Panel A (T vs. S), PC1–PC3 accounted for 49.3%, 18.5%, and 16.8% of the total variance, respectively. While a modest overlap was observed at a discrete coordinate between the T and S groups, the majority of samples exhibited distinct spatial segregation, suggesting a high degree of metabolic concordance alongside residual group-specific variation. In contrast, Panel B (T vs. W) revealed that PC1–PC3 captured 36.5%, 27.9%, and 13.1% of the variance. Here, samples from the T and W groups formed completely discrete clusters with no detectable overlap, demonstrating that GELN intervention elicited a profound systemic metabolic reprogramming distinct from that induced by antioxidant nutrients.

#### 2.3.2. Metabolites Undergo Changes Following the Application of Interventions

**Figure 3:**
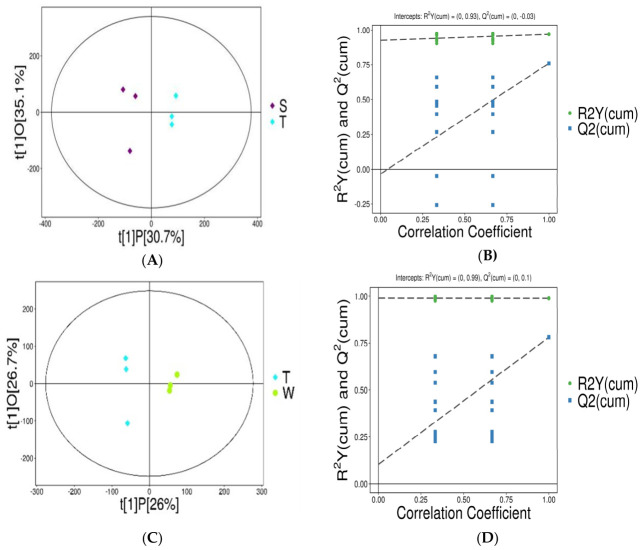
OPLS-DA model scatter plot and permutation test results plot for the OPLS-DA model. (**A**,**B**) represent the comparison between the model control group and the antioxidant nutrient group; (**C**,**D**) represent the comparison between the model control group and the grape exosome group.

To gain deeper insights into intergroup metabolite differences, their correlation with experimental groups, and grape exosome-induced metabolic alterations, we constructed an Orthogonal Partial Least Squares Discriminant Analysis (OPLS-DA) model. Model quality was evaluated via seven-fold cross-validation, and validity was further assessed using cross-validated (explaining variance in the classification variable) and (predictive ability). Permutation testing (iterations) was performed by randomly shuffling to generate null models; none of these iterations showed overfitting in comparisons between the model control group (T group) and either the antioxidant nutrient group (S group) or grape exosome group (W group). OPLS-DA score plots (Figure 3) revealed complete separation of metabolic profiles across all groups. Following differential metabolite identification, we performed multivariate statistical analyses for in-depth data mining. Differential metabolites were defined using thresholds of variable importance in projection (VIP) > 1 and *p* < 0.05. Compared to the T group: We identified 1519 differential metabolites in the S group; 1647 differential metabolites were detected in the W group.

Table 2 (top 10 entries) summarizes differential metabolites between the S and T groups: 264 upregulated and 1255 downregulated. Table 3 (top 10 entries) lists those between the W and T groups: 425 upregulated and 1222 downregulated. Relative to the T group, the W group showed significantly higher riboflavin levels and lower levels of pentadecanoic acid, valproic acid, and isovaleric acid.

#### 2.3.3. Pathway Analysis of Differential Metabolites

**Figure 4:**
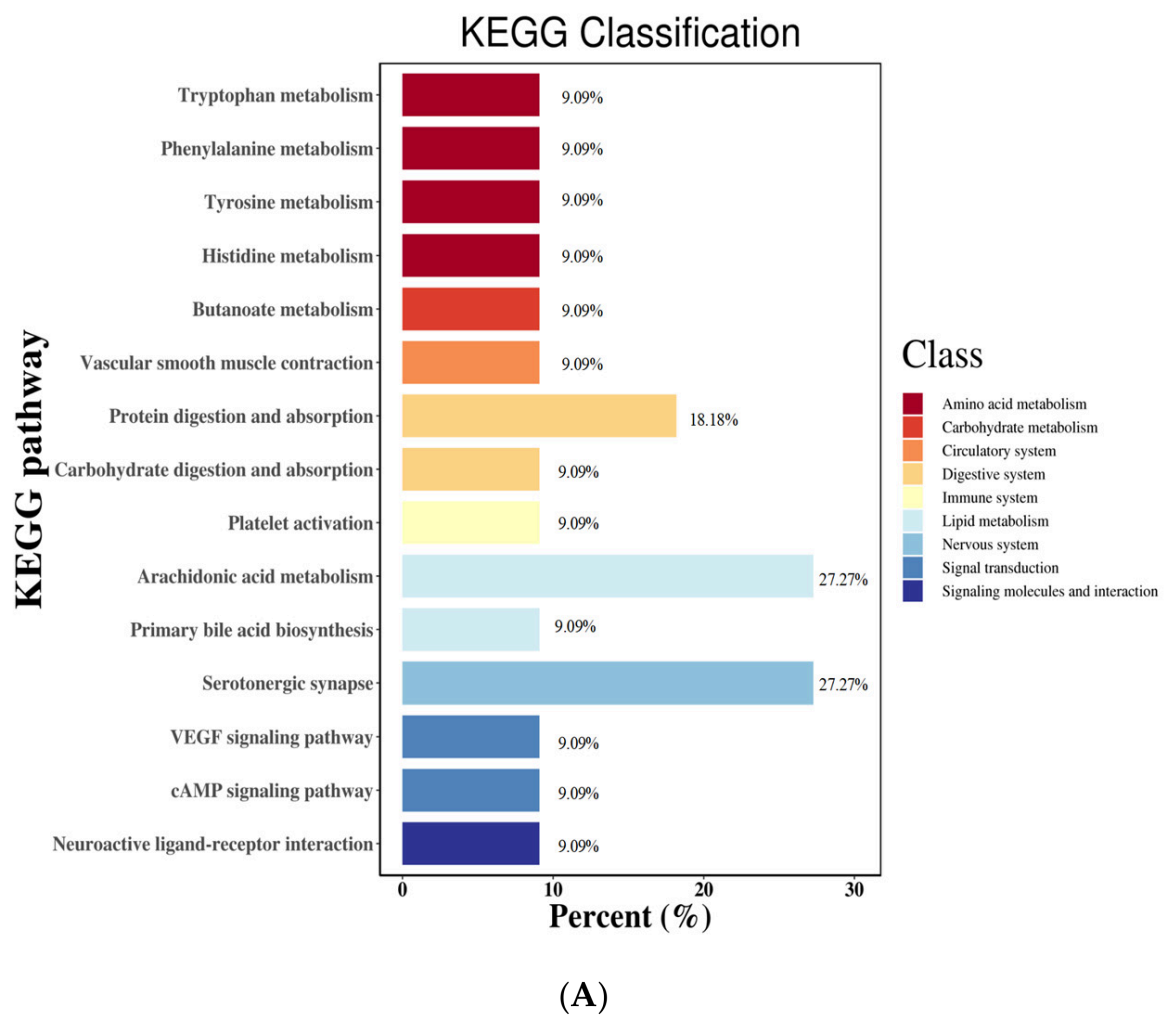

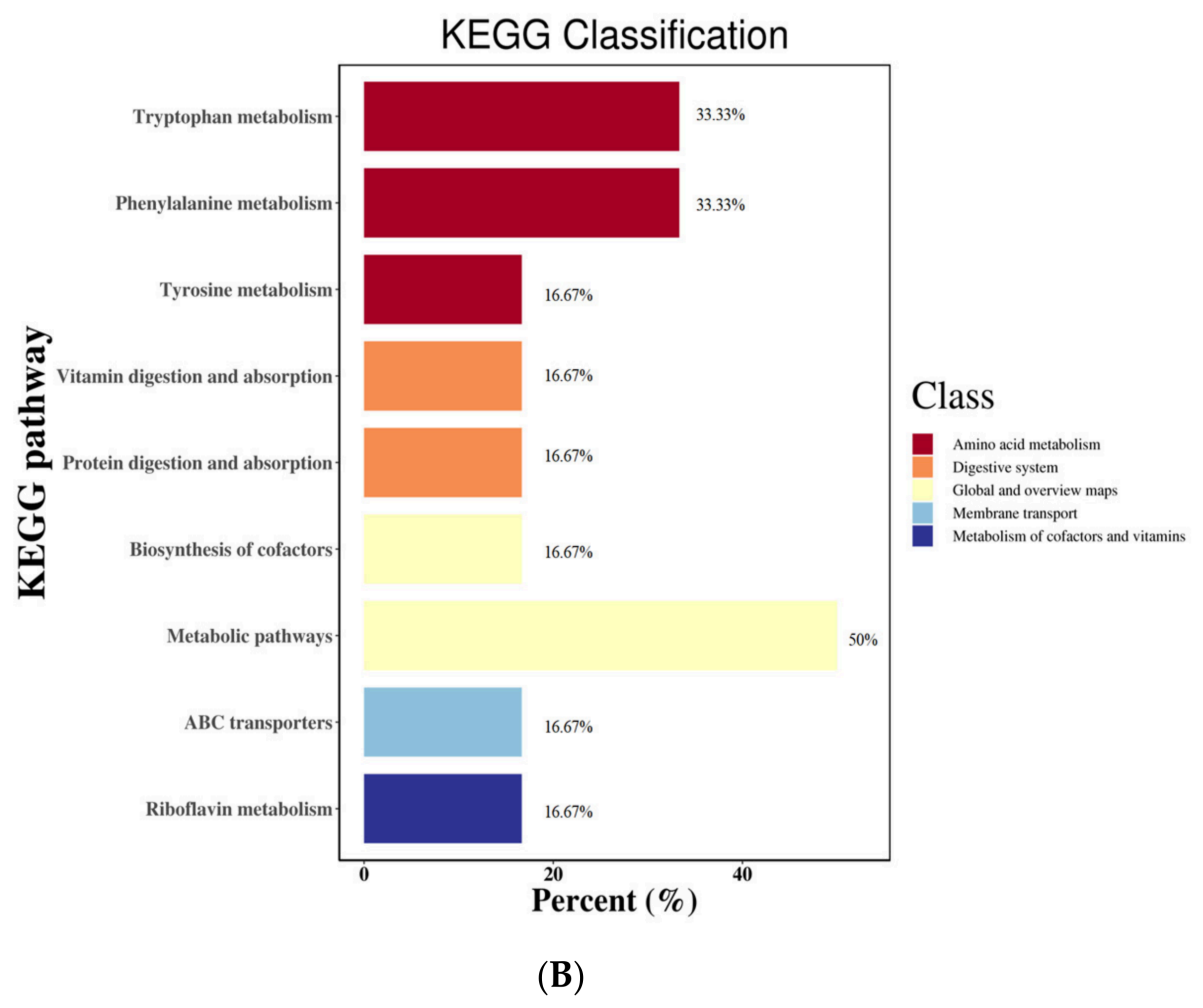
KEGG classification plot of differential metabolites. (**A**) The comparison between the control group and the antioxidant nutrient group; (**B**) represents the comparison between the model control group and the grape exosome group.

To elucidate the metabolic mechanisms underlying the therapeutic effects of grape-derived exosomes on prediabetes, we investigated the specific localization and functional roles of differentially altered metabolites in key metabolic pathways. Pathway enrichment analysis (Figure 4) demonstrated that, compared to the model control group (T group), differential metabolites in the grape exosome-treated group (W group) were predominantly enriched in pathways related to general metabolism, phenylalanine metabolism, and tryptophan metabolism. In contrast, compared to the T group, the antioxidant nutrient-treated group (S group) showed differential metabolites primarily enriched in pathways linked to protein digestion and absorption, arachidonic acid metabolism, and the serotonergic synapse. These findings suggest that the therapeutic effects of grape exosomes on prediabetes may be closely associated with modulation of amino acid metabolism—a pathway uniquely highlighted in the W group but not in the S group.

### 2.4. Targeted Metabolomics Results

#### 2.4.1. Metabolic Principal Component Analysis

**Figure 5:**
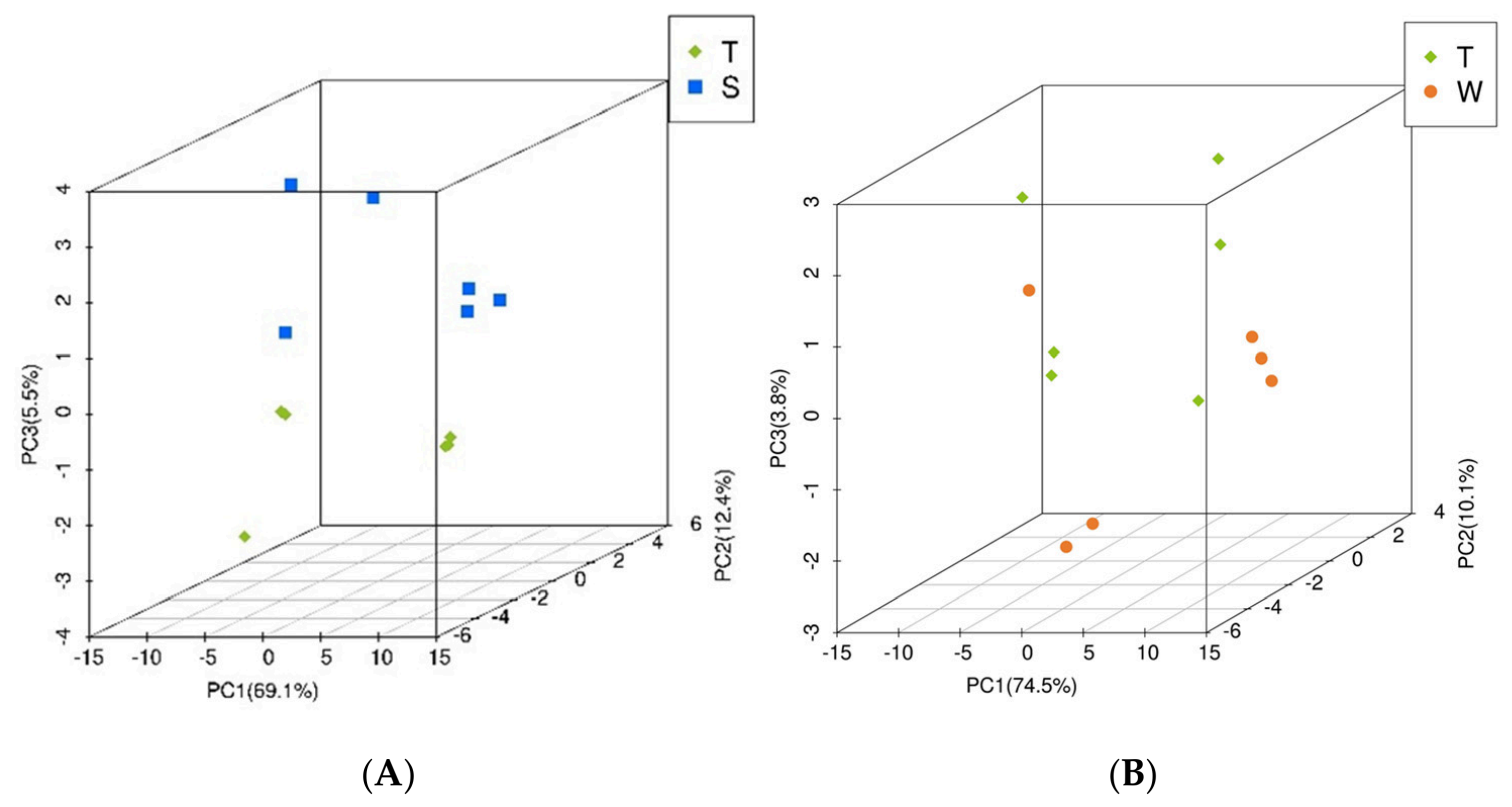
Three-dimensional scatter plot of the PCA model. (**A**) The comparison between the model control group and the antioxidant nutrient group; (**B**) represents the comparison between the model control group and the grape exosome group.

Principal component analysis (PCA) score plots (Figure 5) delineate a clear segregation of metabolic profiles between the model control (T group) and the intervention groups (W [grape exosomes] and S [antioxidant nutrients]). In Panel A (T vs. S), PC1, PC2, and PC3 accounted for 69.1%, 12.4%, and 5.5% of the total variance, respectively, with samples exhibiting non-overlapping spatial distributions. Similarly, Panel B (T vs. W) revealed distinct clustering between the model control and GELN-treated mice, capturing 74.5%, 10.1%, and 3.8% of the variation along the first three components. The pronounced separation observed in both comparisons substantiates that systemic metabolic perturbations induced by the disease model were markedly reprogrammed following antioxidant nutrient or GELN intervention.

#### 2.4.2. Changes in Metabolites Following Intervention

**Figure 6:**
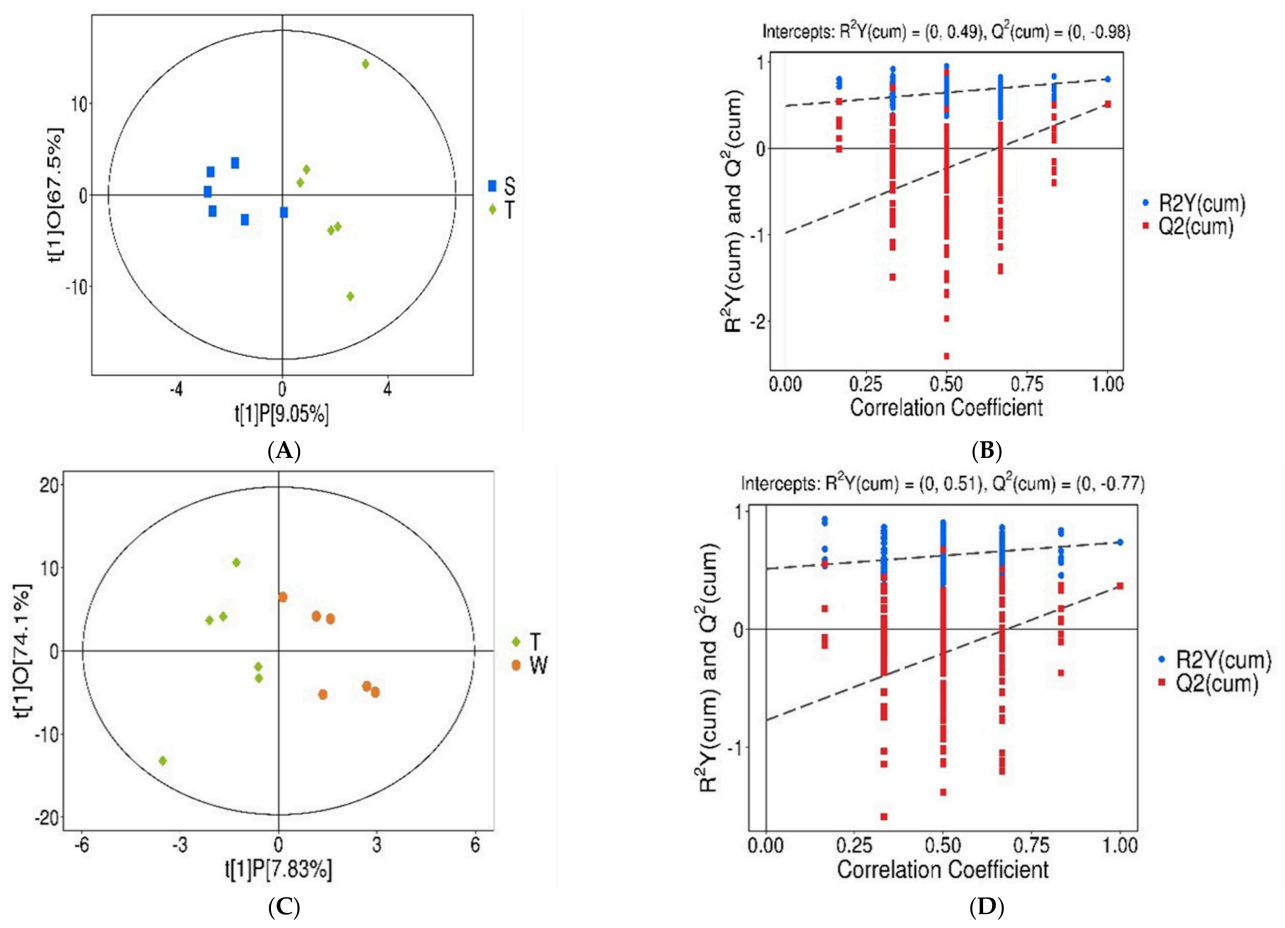

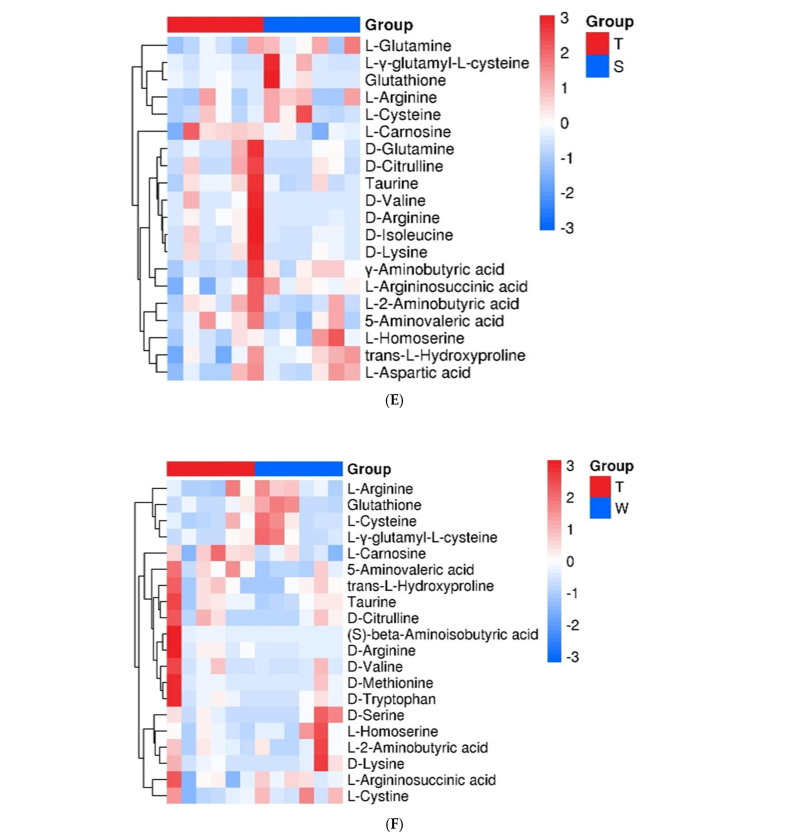
OPLS-DA model scatter plot, permutation test results plot for the OPLS-DA model, and heatmaps of differential metabolites. (**A**,**B**) represent the model control group versus the antioxidant nutrient group; (**C**,**D**) represent the model control group versus the grape exosome group; (**E**) represents the model control group versus the antioxidant nutrient group; (**F**) represents the model control group versus the grape exosome group.

The OPLS-DA score plot (Figure 6) shows clear separation of metabolic profiles across all groups. Results from 200 permutation tests indicated no overfitting in comparisons between the model control group (T group) and either the antioxidant nutrient group (S group) or grape exosome group (W group). Following differential metabolite identification, we performed multivariate statistical analyses to enable in-depth data exploration. Metabolites were screened using thresholds of VIP > 0 and *p* < 1, yielding 64 differential metabolites. Comparisons between the S group and T group identified 16 upregulated and 48 downregulated metabolites (listed in Table 4, top 10). Similarly, comparisons between the W group and T group revealed 23 upregulated and 41 downregulated metabolites (shown in Table 5, top 10). Notably, relative to the T group, the W group showed significantly higher levels of glycine, D-alanine, and L-2-aminobutyric acid—alongside lower levels of ethanolamine, β-alanine, L-alanine, and γ-aminobutyric acid. Heatmaps (Figure 6E,F) depict the relative abundance of the top 10 upregulated and downregulated metabolites.

#### 2.4.3. Pathway Analysis of Amino Acid Changes

**Figure 7:**
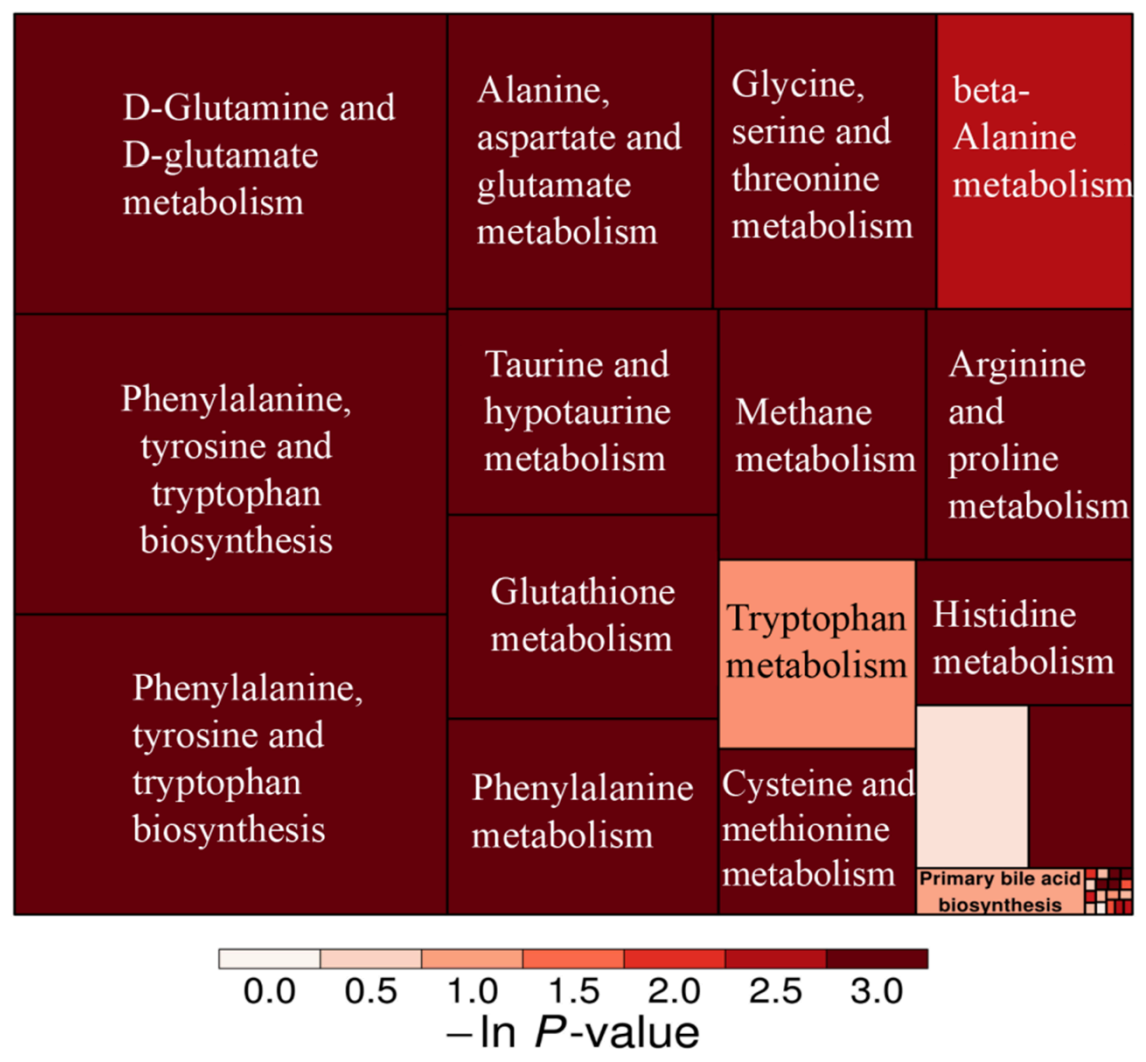
Metabolic pathway analysis chart of metabolites.

To further elucidate how grape-derived exosomes modulate metabolite alterations and their associated pathways, we performed an in-depth analysis of the relevant metabolic networks. Pathway enrichment analysis (Figure 7) identified the most significantly enriched pathways as D-glutamine/D-glutamate metabolism, phenylalanine/tyrosine/tryptophan biosynthesis, and valine/leucine/isoleucine biosynthesis. These results suggest that grape exosomes may alleviate prediabetes by targeting these key metabolic pathways—highlighting their potential as therapeutic candidates for early-stage glucose dysregulation.

To further clarify the direct regulatory relationships between individual key metabolites and specific pathways (avoiding cross-confusion between multiple metabolites and pathways), we summarize the key differential metabolites from both untargeted and targeted metabolomics interventions that meet the statistical screening criteria (untargeted: VIP > 1 and *p* < 0.05; targeted: VIP > 0 and *p* < 1) and are closely associated with prediabetic glucose regulation. These metabolites are organized below in Table 6 according to their “metabolite-pathway” correspondence.

## 3. Discussion

Currently, intervention strategies for prediabetes encompass diverse approaches, with pharmacological interventions gaining increasing prominence. Pharmacotherapy includes both Western medicine and traditional Chinese medicine (TCM). However, conventional Western hypoglycemic agents typically act via single mechanisms and are less effective in preventing pancreatic function deterioration and disease progression—limiting their long-term efficacy for glycemic control. Moreover, prolonged use of certain Western drugs (e.g., glinides, biguanides, thiazolidinediones) is associated with common adverse effects such as hypoglycemia and weight gain [20]. In contrast, TCM interventions for prediabetes are characterized by fewer side effects and greater personalization, making them more suitable for long-term use. Notably, medicinal and edible plant-based foods have emerged as promising candidates: they exert comprehensive synergistic effects on blood glucose and lipid regulation via multiple pathways [21], attracting significant attention in recent years.

In this study, both grape-derived exosome-like nanoparticles (GELNs) and antioxidant nutrient interventions effectively reduced blood glucose levels in prediabetic mice. After 4 and 8 weeks of oral gavage, fasting plasma glucose (FPG) levels in the W group (GELNs) and S group (antioxidant nutrients) were significantly lower than those in the T group (model control). Kumar et al. [22] demonstrated that plant-derived exosome-like nanoparticles (PELNs) improved glucose tolerance in mice by upregulating miR-375 and suppressing aryl hydrocarbon receptor (AhR) expression. Building on this, we hypothesized that GELNs might similarly ameliorate hyperglycemia in diabetic models via analogous mechanisms. Post-intervention, both the W group (GELNs) and S group (antioxidant nutrients) exhibited higher insulin β-cell indices than the T group—indicating that both interventions effectively enhanced pancreatic β-cell function. He et al. [23] reported that mung bean sprout-derived exosome-like nanoparticles (MELNs) improved type 2 diabetes mellitus (T2DM) in mice by activating the PI3K/Akt signaling pathway, upregulating GLUT4/Nrf2/GSK-3β, and reducing GSK-3β levels—thereby boosting antioxidant enzyme activity and alleviating oxidative stress. These findings suggest that plant-derived exosomes modulate insulin signaling and mitigate oxidative stress, collectively contributing to glycemic control. Consequently, we propose that GELNs may enhance pancreatic β-cell function via similar signaling pathways. Nonetheless, the specific grape exosomal miRNAs driving these effects merit further experimental validation.

In this study, we adopted an integrated approach combining untargeted and targeted metabolomics to dissect the potential roles and underlying mechanisms of grape-derived exosomes (GELNs) in diabetes prevention and treatment. Untargeted metabolomics laid the groundwork for subsequent targeted analysis, which validated key findings from the initial screening—enhancing the comprehensiveness and biological relevance of our metabolomic investigation [17].

Untargeted metabolomics analysis revealed that the top 10 differential metabolites following grape exosome intervention (vs. controls) all possessed anti-inflammatory, antioxidant, and metabolic regulatory properties. Following 8 weeks of oral gavage, riboflavin levels in the grape exosome-treated W group were significantly increased. Riboflavin, a water-soluble B vitamin, plays a pivotal role in human metabolism. Prior studies have shown that riboflavin directly impacts diabetes onset, primarily via its antioxidant properties and metabolic regulation. Riboflavin deficiency impairs iron absorption, disrupts tryptophan metabolism, causes mitochondrial dysfunction, and disturbs gastrointestinal/neurological function—ultimately leading to multi-vitamin metabolic disorders and contributing to diabetes development and progression [24]. Riboflavin contributes to energy, carbohydrate, and lipid metabolism via its derivatives: flavin adenine dinucleotide (FAD) and flavin mononucleotide (FMN). Thus, we hypothesize that grape exosome-like nanoparticles (GELNs) may treat type 2 diabetes by replenishing riboflavin or modulating its metabolic pathways. Furthermore, we observed that following 8 weeks of oral gavage administration, the serum levels of pentadecanoic acid, valeric acid, and isovaleric acid were all significantly downregulated in the grape exosome-treated group (W group). The functional implications of these changes are as follows: Pentadecanoic acid, an odd-chain saturated fatty acid, has been previously reported to exert protective effects on insulin sensitivity and metabolic syndrome. Notably, serum levels of odd-chain fatty acids are often elevated in patients with diabetes, suggesting that the downregulation of this metabolite in our study may indicate normalization of lipid metabolic networks [25,26]. Valeric acid serves not only as a critical intermediate in energy metabolism but also participates in bile acid metabolism and the regulation of insulin signaling pathways, potentially mediating indirect effects on glucose homeostasis via modulation of the gut microecology [27,28]. Isovaleric acid, closely associated with amino acid metabolism (particularly branched-chain amino acid metabolism), is frequently elevated in patients with metabolic disorders such as obesity and diabetes. The reduced levels observed in this study may suggest alleviation of abnormal activation of protein degradation pathways [28,29].

Previous research has demonstrated that grape-derived exosome-like nanoparticles (GELNs) modulate diverse metabolic processes via multiple pathways, thereby influencing cellular and organismal health. KEGG enrichment analysis of untargeted metabolomics data revealed that the most significantly altered metabolites in the W group (grape exosome-treated mice) were predominantly enriched in phenylalanine metabolism and tryptophan metabolism. Phenylalanine acts through two key mechanisms: it activates the calcium-sensing receptor (CaSR) on intestinal endocrine cells, increasing glucagon-like peptide-1 (GLP-1) secretion [30], and it is converted to dopamine—which then activates dopamine receptors to enhance insulin secretion [31,32]. Tryptophan, an essential amino acid, is metabolized by gut microbiota into bioactive molecules including indole and its derivatives: indole-3-lactic acid (ILA), indole-3-propionic acid (IPA), and indole-3-aldehyde (IAld) [33]. Tryptophan metabolites are increasingly implicated in type 2 diabetes mellitus (T2DM) pathogenesis [34,35]:

- Indole stimulates intestinal L cells to secrete GLP-1, promoting insulin release and lowering blood glucose;
- IPA acts via the aryl hydrocarbon receptor (AhR) [36,37] to exert anti-inflammatory and cytoprotective effects. In vivo studies show indole reduces hepatic steatosis and inflammation in high-fat diet-fed rats, while in vitro work confirms it decreases lipid accumulation and modulates inflammatory responses [38].

Beyond these, AhR activation by tryptophan metabolites regulates diverse physiological processes—including immune responses, inflammation, and cellular differentiation [39]—highlighting the pleiotropic roles of tryptophan-derived molecules in metabolic health.

Non-targeted metabolomics analysis revealed that grape exosome intervention induced significant changes in the levels of differential metabolites—including riboflavin, pentadecanoic acid, valeric acid, and isovaleric acid—in prediabetic mice. These changes were tightly associated with riboflavin metabolism, phenylalanine/tryptophan metabolism, and linked amino acid pathways: Riboflavin (vitamin B_2_), a critical cofactor for flavoproteins (e.g., mitochondrial complex I/II, glutathione reductase), directly participates in energy metabolism (tricarboxylic acid cycle, oxidative phosphorylation) and antioxidant defense (glutathione regeneration), serving as a linchpin for basal metabolic homeostasis [40]. Pentadecanoic acid, a long-chain saturated fatty acid, reflects the reprogramming of the lipid metabolic network (balance between lipogenesis and lipolysis). Prediabetes is characterized by lipid overload (excessive lipolysis, increased hepatic de novo lipogenesis), and its downregulation indicates reduced lipid metabolic burden [41,42]. Isovaleric acid, a catabolic intermediate of branched-chain amino acids (BCAAs, e.g., valine, isoleucine), also acts as a cross-node in the aromatic amino acid (AAA, e.g., phenylalanine, tryptophan) metabolic network. Its altered level suggests coordinated reprogramming of phenylalanine/tryptophan and BCAA metabolism [43,44].In summary, the dynamic fluctuations of these metabolites demonstrate that grape exosomes achieve holistic regulation of energy, lipid, and amino acid metabolic networks via multi-pathway synergy.

In targeted metabolomics analysis, we observed a significant elevation in glycine levels in the grape exosome-treated mouse group (W group). Numerous studies have established a negative correlation between glycine and both prediabetes and type 2 diabetes (T2D) [45]. Furthermore, glycine supplementation enhances insulin responsiveness and glucose tolerance [46,47]—with circulating glycine modulating multiple metabolic processes and improving glucose homeostasis upon supplementation [48]. Current evidence indicates that glycine acts through dual pathways: centrally via the dorsal vagal complex’s N-methyl-D-aspartate (NMDA) receptor and systemically via glycine receptors—to reduce oxidative stress, dampen inflammation, and boost insulin secretion from pancreatic islets [48]. Additionally, glycine directly stimulates insulin release from β-cells by engaging their surface glycine receptors [48].

Targeted metabolomics revealed that both the grape exosome (W) and antioxidant nutrient (S) groups in mice showed significant enrichment of differential metabolites in pathways for valine/leucine/isoleucine biosynthesis and D-glutamine/D-glutamate metabolism. Bridget et al. [49] demonstrated via leucine supplementation in animal models that leucine improves glucose regulation—specifically fasting blood glucose and oral glucose tolerance—and enhances pancreatic insulin secretion. This suggests leucine acts as a potential insulinotropic agent to improve glucose homeostasis, though its underlying mechanisms remain unclear. Similarly, Bahaguli et al. [50] reported a negative correlation between circulating valine/leucine/isoleucine levels and type 2 diabetes (T2D) incidence. As branched-chain amino acids (BCAAs), valine/leucine/isoleucine are metabolized to α-keto acids and then to acetyl-CoA derivatives—serving as critical nutritional signals that boost insulin secretion from pancreatic β-cells. These amino acids also regulate satiety and glucose metabolism via both peripheral and central pathways [51]. Reduced isoleucine/leucine/valine levels indicate impaired BCAA biosynthesis, which directly suppresses insulin secretion. Additionally, glutamine modulates the PI3K/AKT/GLUT4 signaling pathway to exert hypoglycemic effects [52].

In this study, targeted metabolomics analysis revealed that grape exosome intervention induced significant remodeling of multiple amino acid levels in prediabetic mice: glycine, D-alanine, and L-2-aminobutyric acid levels increased, whereas ethanolamine, β-alanine, and GABA levels decreased. These changes may be closely associated with three core metabolic pathways: D-glutamine/D-glutamate metabolism, phenylalanine/tyrosine/tryptophan biosynthesis, and valine/leucine/isoleucine biosynthesis. Glycine, a core node of the glutamate/GABA metabolic network, may reflect the rebalancing of glutamate metabolism—enhancing antioxidant capacity (e.g., promoting glutathione synthesis), regulating one-carbon metabolism (e.g., providing methyl donors), and activating insulin signaling pathways (e.g., Akt), thereby directly improving metabolic status [53]. Although the phenylalanine/tyrosine/tryptophan pathway primarily relies on exogenous intake, its downstream products (e.g., 5-hydroxytryptamine (5-HT), nicotinamide adenine dinucleotide (NAD^+^)) can engage in cross-talk with glycine metabolism, antioxidant systems, and energy regulatory networks, forming metabolic synergy [54]. The upregulation of D-alanine may be linked to D-glutamate metabolism and D-amino acid metabolic activities derived from gut microbiota—gut microbiota are key producers of D-amino acids, and changes in their metabolic activity may modulate host D-alanine levels via microbiota-host interaction [55]. The decrease in GABA levels may indicate the recovery of islet β-cell regulatory function and the restoration of neuro-metabolic homeostasis. As an inhibitory neurotransmitter in islet β-cells, reduced GABA may normalize insulin secretion rhythm (alleviating excessive inhibition under hyperglycemia) [56]. L-2-aminobutyric acid, sharing structural homology with branched-chain amino acids (BCAAs, e.g., valine, leucine, isoleucine), may indirectly associate with BCAA biosynthesis pathways. Given that BCAA metabolic disorders are a hallmark of insulin resistance, changes in their metabolic bypass may suggest the body’s adaptive response to metabolic stress (e.g., diverting toxic intermediates, optimizing nitrogen metabolism) [57,58]. In summary, these dynamic changes in amino acid levels reveal that grape exosomes may improve glucose and lipid metabolism through multi-pathway synergistic regulation: targeting amino acid metabolic nodes (e.g., glycine) and optimizing core pathways (e.g., BCAA biosynthesis), ultimately mitigating pathological features of prediabetes such as insulin resistance and lipid overload.

Despite observing significant elevations in glycine and riboflavin levels post-GELNs intervention, and given their established correlations with improved insulin sensitivity, these changes may not be directly mediated by GELNs but rather reflect secondary compensatory responses to restored metabolic status. For example, population studies demonstrate that improvements in glycemic control precede rises in glycine levels following insulin sensitizer treatment (e.g., pioglitazone), suggesting glycine elevation is a consequence—not a driver—of metabolic repair [59]. Mechanistically, insulin signaling pathways (e.g., AKT-FOXO1) may directly upregulate hepatic glycine synthase (AGXT) [60] expression to promote endogenous glycine synthesis, while increased FAD/FMN consumption could feedback to upregulate riboflavin transporters RFVT2/3 [61], elevating riboflavin levels. Thus, these metabolites are more likely “accompanying biomarkers” of metabolic improvement than “direct effectors” of GELNs’ functions. Future studies using isotope tracing, gene editing, or exogenous metabolite supplementation are required to validate their causal relationship with GELNs intervention and clarify their genuine roles in glucose and lipid metabolism regulation.

Corroborating our initial hypothesis, integrated physiological assessments coupled with untargeted and targeted metabolomics analyses demonstrated that GELNs effectively mitigate prediabetic pathophysiology. Mechanistically, GELNs markedly attenuated fasting hyperglycemia and preserved pancreatic β-cell function in prediabetic mice. Furthermore, GELNs orchestrated a comprehensive remodeling of the intestinal metabolome, characterized by the upregulation of protective metabolites such as riboflavin and glycine. Pathway analysis revealed significant modulation of pivotal metabolic cascades, including branched-chain amino acid (BCAA) biosynthesis, phenylalanine and tryptophan metabolism, and the glutamine-glutamate cycle. Collectively, these phenotypic and metabolomic findings establish that the anti-prediabetic efficacy of GELNs is mediated through the systematic orchestration of key metabolic networks, thereby validating our central hypothesis.

Nonetheless, this study is subject to two principal experimental constraints that warrant explicit acknowledgment. First, the induction of a prediabetic, insulin-resistant phenotype in all experimental cohorts was not complemented by a lean, non-diabetic control group; this omission constrains our capacity to delineate the comparative physiological metabolic profiles between homeostatic and pathophysiological states. Second, the absence of a sonicated GELN cohort precludes a definitive assessment of whether the structural integrity of these nanoparticles is a prerequisite for the observed metabolic amelioration. Consequently, this limits the in vivo evidence substantiating the regulatory role of encapsulated miRNAs. Subsequent investigations will integrate both healthy control cohorts and mechanistically disrupted (sonicated) GELN groups to bridge these gaps and deepen our understanding of the underlying mechanisms. Collectively, these refinements will fortify the translational validity of GELNs as a viable therapeutic candidate against prediabetes.

## 4. Materials and Methods

### 4.1. Main Experimental Reagents

Streptozotocin (STZ, Cat. No. S0130, Sigma-Aldrich, St. Louis, MO, USA); Sodium citrate (Sigma-Aldrich, USA); Vitamin E (Shanghai Biochemical Co., Ltd., Shanghai, China); Sodium selenite pentahydrate (NaSeO_3_·5H_2_O, AR grade, Tianjin Institute of Chemical Reagents, Tianjin, China); Vitamin C (Desmann Jiangshan Pharmaceutical (Jiangsu) Co., Ltd., Jingjiang, China); Grape-derived exosome-like nanoparticles (Wuhan Jinkairui Bioengineering Co., Ltd., Wuhan, China); Phosphate Buffered Saline (PBS, Hyclone, Logan, UT, USA); 4% paraformaldehyde solution (Shanghai Baishark Biotechnology Co., Ltd., Shanghai, China); glucose (Aladdin, Shanghai, China); Insulin powder (Sigma-Aldrich, USA); Triglyceride (TG) assay kit (Nanjing Jiancheng Bioengineering Institute, Nanjing, China); Total Cholesterol (TC) assay kit (Nanjing Jiancheng Bioengineering Institute, China); Mouse Insulin (INS) ELISA kit (Shanghai Youxuan Biotechnology Co., Ltd., Shanghai, China).

### 4.2. The Extraction of Grape Exosome-like Nanoparticles

This study centered on grape-derived exosome-like nanoparticles (GELNs) from Xinjiang Manai grapes. GELNs were isolated via ultracentrifugation and prepared as a 0.5 mg/mL suspension in phosphate-buffered saline (PBS), following a previously reported methodology [62]. 

The extraction protocol was as follows:

(1) Sample Preprocessing: Grapes were weighed, peeled, and reweighed. After rinsing with ultrapure water, they were juiced with PBS, filtered through gauze, and centrifuged at 1000× *g* for 10 min to collect the supernatant. The supernatant was further centrifuged at 3000× *g* for 30 min (retaining the supernatant), concentrated using a 100 kDa ultrafiltration tube (3500 rpm, 30 min), and then centrifuged at 10,000× *g* for 30 min. The final supernatant was filtered through a 0.22 μm microporous membrane.

(2) Exosome Isolation via Ultracentrifugation: The processed sample was centrifuged at 100,000× *g* for 1 h (4 °C), and the supernatant was discarded. The pellet was resuspended in PBS and ultracentrifuged again at 100,000 × *g* for 1 h (4 °C). After removing the supernatant, the pellet was resuspended in PBS to isolate GELNs. An additional low-speed centrifugation step clarified the final supernatant.

PBS was adopted during grape juicing, pellet resuspension after ultracentrifugation, and subsequent oral gavage treatment. When mixed with grape pulp during juicing, PBS stabilizes pH and osmotic pressure to shield vesicles from osmotic shock and membrane protein denaturation. It resuspends centrifugal pellets to preserve intact vesicle structure and prevent nanoparticle aggregation. Furthermore, PBS acts as an isotonic carrier for oral administration to protect GELNs in the gastrointestinal environment.

**Figure 8:**
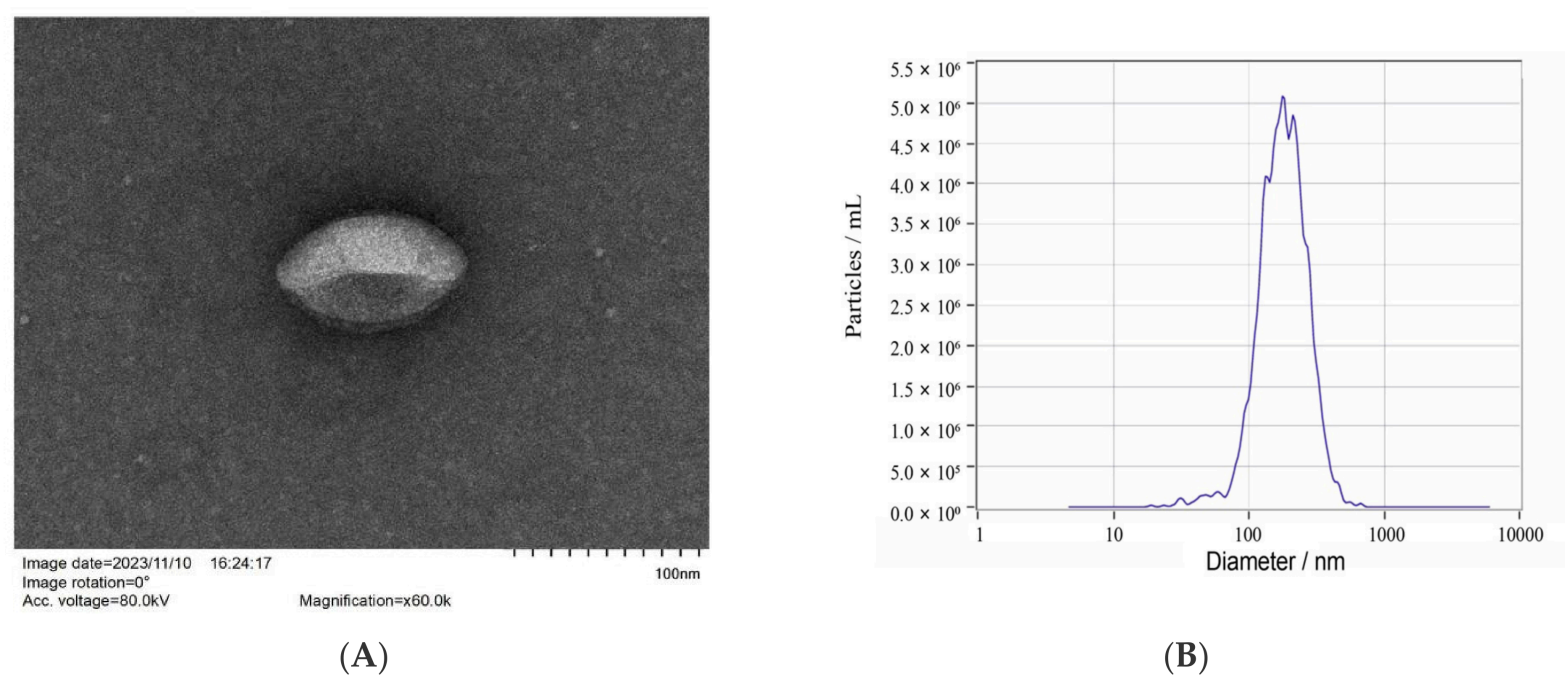
TEM image of GELNs and a schematic diagram of GELNs size and concentration. Note: (**A**). TEM image of GELNs. (**B**). Nanoparticle tracking analysis (NTA) profile of grape-derived exosome-like nanoparticles (GELNs). The *X*-axis represents the particle diameter (nm) on a logarithmic scale, the *Y*-axis indicates the particle concentration (particles per milliliter), and the blue curve shows the particle size distribution of isolated GELNs.

Transmission electron microscopy (TEM; 80 kV) of GELNs (Figure 8A) revealed irregularly round/elliptical structures with a central concavity and dark membranous boundary—consistent with the typical cup-shaped exosome morphology. Nanoparticle tracking analysis (NTA; Figure 8B) showed an average diameter of 193 nm and a concentration of 4.1 × 10^11^ particles/mL. Bicinchoninic acid (BCA) assay measured the protein concentration of the vesicles at 28.39 μg/μL.

GELNs were administered orally at a dose of 0.5 mg/mL in PBS, following the protocol established by Rasoul Saleh et al. [63].

### 4.3. miRNA Profiles and Expression Differences in Grape-Derived Exosome-like Nanoparticles (Gelns)

**Figure 9:**
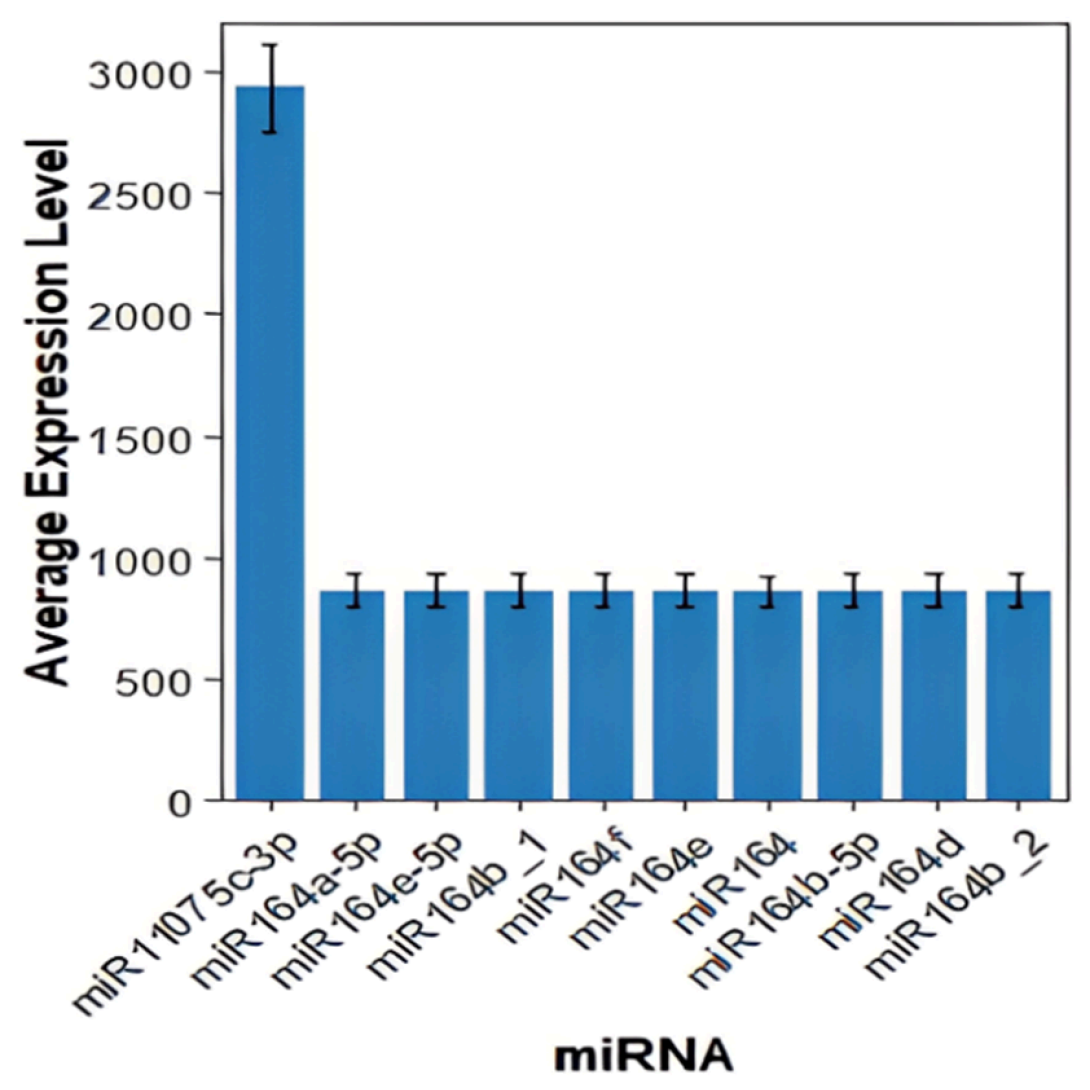
Expression levels of the top 10 miRNAs in the exosome-like nanoparticles of mare’s milk grape. Note: Top 10 abundant miRNAs in GELNs. Comparable column heights of miR164 subtypes are due to their highly consistent expression abundance.

Small RNA sequencing revealed that miR11075c-3p was the most abundant miRNA in GELNs, with a read count of 8804 (Figure 9). This was followed by members of the miR164 family (miR164a-5p and miR164e-5p), with 2604–2606 reads. All nine identified miR164 homologs display exceptional sequence conservation and virtually identical transcript abundances in GELNs, culminating in uniform column heights within the quantitative histogram. The high abundance of miR11075c-3p suggests it may play a key role in GELN-mediated metabolic regulation, although its function remains unreported. In contrast, the miR164 family has been shown to target IRS1 and modulate insulin signaling [64]. 

### 4.4. Prediction and Functional Analysis of Human Target Genes for Vvi-miRNAs

**Figure 10:**
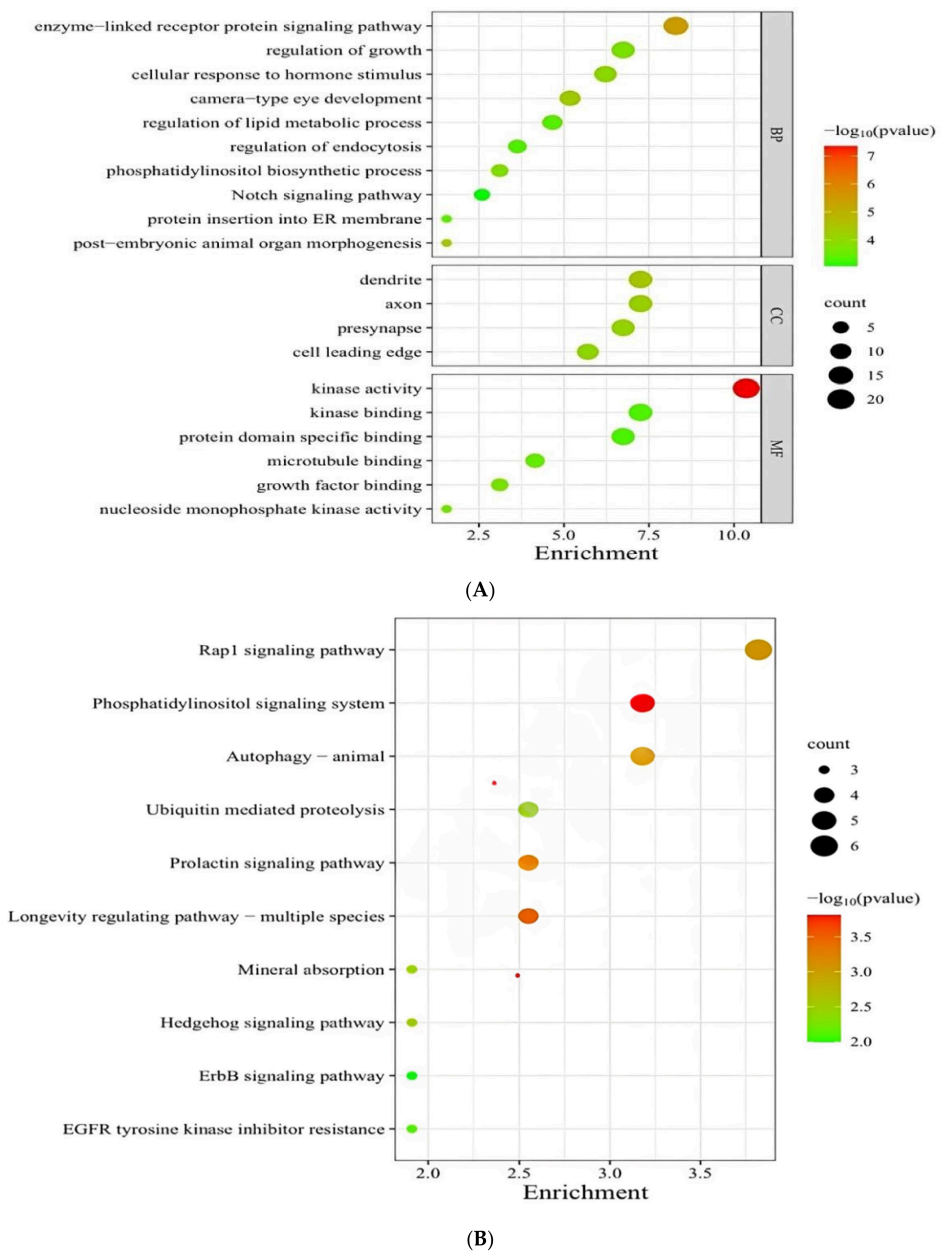
GO enrichment analysis diagram and KEGG enrichment analysis diagram of vvi-miRNA gene. Note: (**A**) is the GO enrichment analysis diagram, and (**B**) is the KEGG enrichment analysis diagram.

First, we predicted potential human target genes for vvi-miRNAs by performing complementary sequence matching using the online psRNATarget database, yielding 615 candidate targets. After removing duplicates, 157 unique target genes were retained. These genes were then submitted to the Metascape database for Gene Ontology (GO) functional enrichment analysis and Kyoto Encyclopedia of Genes and Genomes (KEGG) pathway enrichment analysis to elucidate the biological processes and signaling pathways in which they are involved. GO enrichment analysis revealed that the target genes were primarily enriched in biological processes such as “enzyme activity activation” and “enzyme-linked receptor protein signaling” (Figure 10A). KEGG pathway analysis further indicated significant enrichment in key pathways including the “phosphatidylinositol signaling system” and “RAP1 signaling pathway” (Figure 10B). Finally, we screened for key target genes using the STRING database and evaluated their network importance via the Betweenness algorithm, identifying the top 10 target genes (Table 7). Among these, the ITCH gene exhibited the highest score, suggesting it may serve as one of the core target genes of vvi-miRNAs.

### 4.5. Preparation of Antioxidant Nutrients

The selection of antioxidant nutrients was guided by three compounds—vitamin C (VC), vitamin E (VE), and selenium (SE)—which had previously demonstrated strong performance in an antioxidant dietary pattern identified by our research team [19]. Doses were determined by referencing the 2023 Chinese Dietary Nutrient Reference Intakes (DRIs), a standard published by the Chinese Nutrition Society. For adults, the DRIs recommend 100 mg/day for vitamin C, 14 mg/day for vitamin E, and 60 μg/day for selenium. These human-equivalent doses were converted to mouse doses using a species-specific conversion factor of 9.01, yielding final intervention doses of 15.17 mg/kg for VC, 2.12 mg/kg for VE, and 9.1 μg/kg for SE. All compounds were dissolved in phosphate-buffered saline (PBS) and administered via daily oral gavage, with dosing adjusted to each mouse’s body weight.

### 4.6. Animal Selection and Sample Acquisition

Eighteen male SPF-grade C57BL/6J mice were procured and housed in groups of six per cage in an SPF animal facility. Mice were acclimated to a standard chow diet for 7 days to adapt to the new environment. At week 0, body weights were recorded, and baseline fasting blood glucose (FBG) levels were measured.

The model was established using a protocol adapted from Wang Shaoyun et al. [65] (supported by the “13th Five-Year” National Key R&D Program). Mice were maintained at 20–25 °C with 45–65% relative humidity and fed a high-fat diet (60% kcal from fat) for 3 weeks. After a 12-h fast, mice received an intraperitoneal injection of freshly prepared streptozotocin (STZ; 60 mg/kg) in light-protected ice-cold saline (administered within 30 min of preparation). Oral glucose tolerance tests (OGTT) were performed on days 7 and 21 post-STZ injection by collecting tail vein blood to measure glucose levels. Prediabetes was preliminarily defined as FBG < 7.8 mmol/L and 2-h postprandial glucose (2h-PG) between 7.8 and 11.1 mmol/L [66]. Mice failing to meet these criteria received additional STZ doses until the model criteria were satisfied. Post-modeling, all groups except the control continued on the high-fat diet and received their respective interventions.

The combined high-fat diet and 60 mg/kg STZ injection protocol used in this study could induce obvious insulin resistance and relatively severe prediabetic glucose metabolic disorders, which is suitable for evaluating the intervention effects of test substances on obvious glucose intolerance.

Prediabetic mice were randomly divided into three groups (n = 6 per group): (a) Model control (T group): No treatment; (b) Grape-derived exosome-like nanoparticle (W group): Daily oral gavage of GELNs; (c) Antioxidant nutrient (S group, positive control): Daily oral gavage of antioxidant nutrients. Fresh fecal samples were collected 2 days prior to euthanasia. Following the 8-week gavage regimen, mice were fasted for 12 h (ad libitum access to water). Blood was obtained via retro-orbital venipuncture under anesthesia, after which mice were euthanized. Fresh cecal and partial small intestinal contents were harvested for downstream analysis.

**Figure 11:**
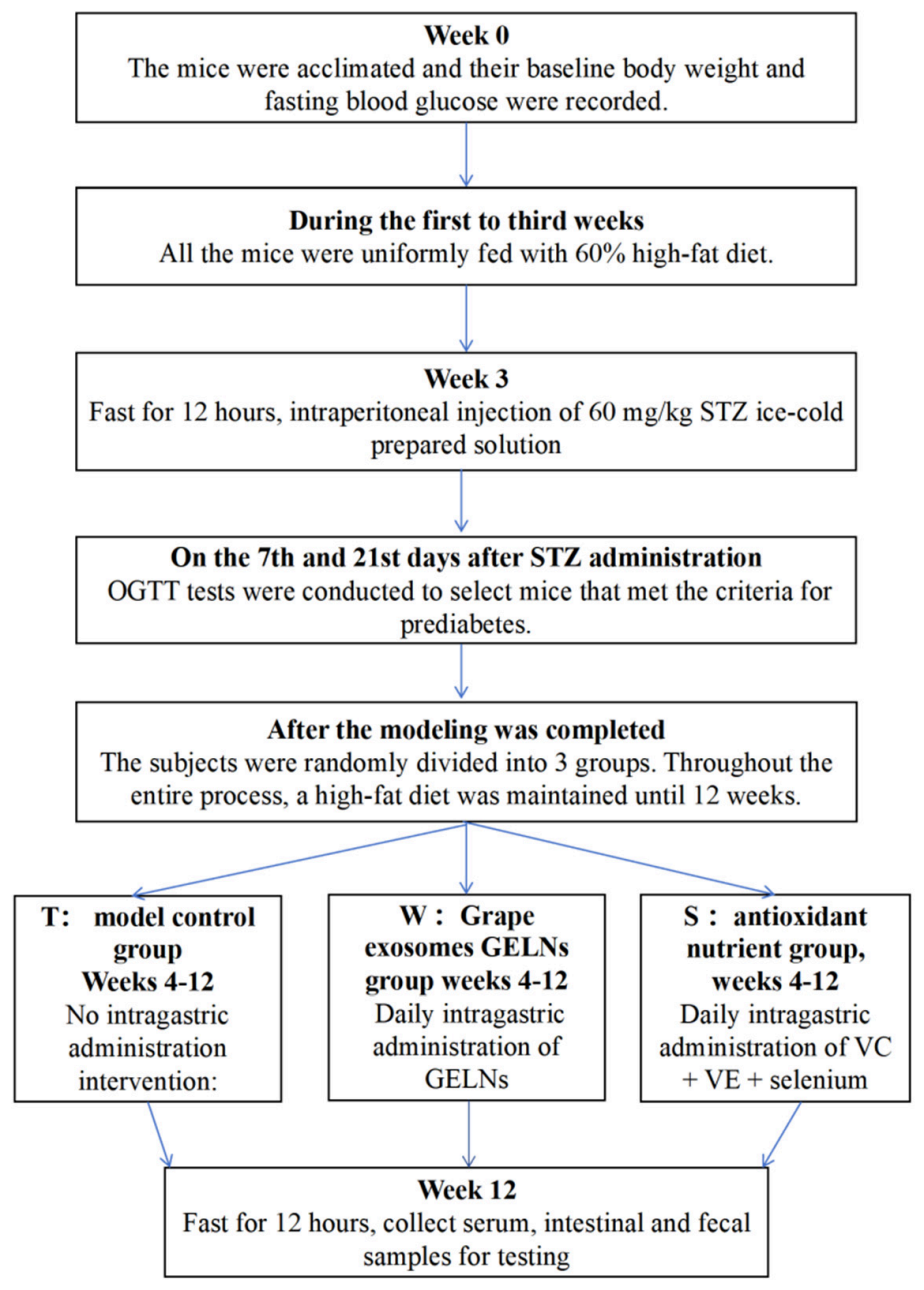
Flowchart of intervention measures implementation.

The specific intervention process can be seen in the flowchart below (Figure 11).

### 4.7. Measurement of Serum Insulin, Serum Total Cholesterol (Tc) Content, and the Insulin Beta-Cell Index

Serum insulin levels were quantified via enzyme-linked immunosorbent assay (ELISA) using a two-antibody, one-step sandwich format. Serum total cholesterol (TC) concentration was measured according to the manufacturer’s protocol for the assay kit. Homeostatic Model Assessment of β-cell function (HOMA-β) was calculated using the formula:

$$HOMA-\beta =\frac{20\;X\;fastinginsulin}{\left(fastingglucose-3.5\right)}$$

where glucose is expressed in mmol/L.

### 4.8. Metabolomics Testing

For metabolomic analysis, 25 mg of sample was precisely weighed into a 4 °C EP tube. Homogenization beads were added alongside 500 μL of extraction solvent—comprising methanol:acetonitrile:water (2:2:1, *v*/*v*/*v*)—preloaded with isotopically labeled internal standards. Samples were vortexed for 30 s, followed by homogenization at 35 Hz for 4 min. This was followed by three cycles of ice-water bath sonication (5 min/cycle). The mixture was then incubated at −40 °C for 1 h and centrifuged at 12,000× *g*(4 °C) for 15 min to precipitate impurities. Supernatant was carefully transferred to sampling vials for instrumental analysis, with aliquots reserved as quality control (QC) samples.

Polar metabolites were separated using a Thermo Fisher Vanquish ultra-high-performance liquid chromatography (UHPLC) system equipped with a Waters ACQUITY UPLC BEH Amide column (2.1 mm × 100 mm, 1.7 μm particle size). The mobile phase consisted of:

Solvent A: 25 mmol/L ammonium acetate + 25 mmol/L ammonia in water;

Solvent B: Acetonitrile.

Sample tray temperature was maintained at 4 °C, and the injection volume was 2 μL.

Non-polar metabolites were analyzed using the same UHPLC system paired with a Phenomenex Kinetex C18 column (2.1 mm × 100 mm, 2.6 μm particle size). Mobile phases were adjusted to:

Solvent A: 0.01% (*v*/*v*) acetic acid in water;

Solvent B: Isopropanol:acetonitrile (1:1, *v*/*v*).

Sample tray temperature remained at 4 °C, with an injection volume of 2 μL for all analyses.

### 4.9. Data Processing and Statistical Analysis

Quantitative data are presented as mean ± standard deviation (SD). For intergroup comparisons of continuous variables: Parametric data (meeting assumptions for ANOVA) underwent one-way analysis of variance (ANOVA); Non-parametric data were analyzed using the Kruskal–Wallis (K-W) test.

Statistical significance was defined as *p* < 0.05 (α = 0.05). Graphs (bar charts, line plots) were generated using GraphPad Prism 10.1.2, and all statistical tests were performed with SPSS 26.0.

The foundational analyses performed on metabolomics data included clustering analysis, metabolite classification statistics, multivariate principal component analysis (PCA), screening and identification of differential metabolites, univariate t-test statistical analysis, and orthogonal partial least squares discriminant analysis (OPLS-DA). Advanced analyses comprised hierarchical clustering of differential metabolites, KEGG annotation and enrichment analysis of differential metabolites, and metabolic pathway analysis.

## 5. Conclusions

In this study, we integrated untargeted and targeted metabolomics to dissect the roles and underlying mechanisms of grape-derived extracellular vesicles (GELNs) in diabetes prevention and treatment. Our findings demonstrate that both GELN and antioxidant nutrient interventions significantly reduced blood glucose levels in prediabetic mice—via modulation of key metabolic pathways including phenylalanine metabolism, tryptophan metabolism, branched-chain amino acid (BCAA) biosynthesis, and D-glutamine/D-glutamate metabolism—thereby contributing to improved cellular and organismal health.

Mice treated with GELNs (W group) exhibited a marked elevation in riboflavin levels, a metabolite critically linked to diabetes pathogenesis due to its essential role in human metabolism (e.g., energy production, antioxidant defense). Furthermore, tryptophan-derived metabolites, BCAAs, and glutamine were identified as pivotal regulators of immune responses, inflammation, cellular differentiation, and insulin secretion—processes central to T2DM pathophysiology.

Collectively, these results suggest that GELNs likely exert therapeutic effects on T2DM by replenishing key metabolites (e.g., riboflavin, BCAAs) or modulating their associated pathways. This work provides novel mechanistic insights and potential therapeutic avenues for diabetes management, highlighting the Value of GELNs as a natural, multi-target intervention for metabolic disease.

This study still has some limitations. For instance, it did not provide direct evidence at the enzyme expression level and tissue uptake level. However, we have retained samples as much as possible within the ethical scope. Future research will plan multi-time-point sampling and fluorescence labeling experiments in advance to improve them through fluorescence tracing and enzymatic detection.

## Figures and Tables

**Table 1:** Effects of intervention strategies on FINS, HOMA-IR, and HOMA-β in mice with prediabetes ($\bar{x}$ ± s).

	Control Group (n = 6)	Model Group (n = 6)	GELNs Group (n = 6)	Nutrient Group (n = 6)	F	*p*
FINS (ng/mL)	2.56 ± 0.19	2.25 ± 0.09 ^a^	2.37 ± 0.17 ^a^	2.22 ± 0.09 ^a^	7.147	0.002
HOMA-IR	23.62 ± 2.78	30.94 ± 17.99	19.76 ± 5.02 ^b^	19.30 ± 2.68 ^b^	5.435	0.008
HOMA-β	256.74 ± 33.22	162.06 ± 73.74	332.13 ± 171.43	258.03 ± 70.36	2.851	0.063

a: denotes a statistically significant difference in comparison to the Control Group (*p* < 0.05); b: denotes a statistically significant difference in comparison to the Model Group (*p* < 0.05); c: denotes a statistically significant difference in comparison to the Nutrient Group (*p* < 0.05).

**Table 2:** Results of differential metabolite screening between the antioxidant nutrient group and the model control group (top 10 rows).

MS2 Name	MS2 Score	VIP	*p*-Value	FOLD CHANGE	LOG_ FOLDCHANGE
↓Valeric acid	3.990	5.172	0.022	0.005	−7.794
↓Isovaleric acid	3.980	5.172	0.022	0.005	−7.794
↓Butanoic acid	3.960	1.180	0.037	0.165	−2.601
↓7alpha,25-Dihydroxycholesterol	3.950	1.227	0.006	0.171	−2.548
↓4-Hydroxyphenylacetic acid	3.940	2.747	0.001	0.140	−2.835
↑2-oxindole-3-acetate	3.930	1.077	0.005	4.758	2.250
↑N-Acetylhistamine	3.920	1.909	0.000	18.941	4.243
↓Hexadecatrienoylcarnitine	3.900	1.263	0.003	0.154	−2.702
↓Arachidonoylcarnitine (Car(20:4))	3.890	1.191	0.009	0.183	−2.454
↓Prostaglandin B2 (PGB2)	3.890	1.068	0.004	0.190	−2.397

**Table 3:** Results of differential metabolite screening between the grape exosome group and the model control group (top 10 rows).

MS2 Name	MS2 Score	VIP	*p*-Value	FOLD CHANGE	LOG_ FOLDCHANGE
↓Pentadecanoic acid	3.990	1.029	0.022	0.311	−1.684
↓Valeric acid	3.990	1.829	0.038	0.326	−1.619
↓Isovaleric acid	3.980	1.829	0.038	0.326	−1.619
↑Riboflavin	3.970	1.029	0.037	2.560	1.356
↓LPE(14:0)	3.950	1.398	0.017	0.280	−1.836
↓Azelaic acid	3.940	1.028	0.014	0.293	−1.771
↓4-Hydroxyphenylacetic acid	3.940	1.348	0.012	0.306	−1.706
↓Kynurenic acid	3.930	1.245	0.003	0.237	−2.078
↓Phenylacetylglycine	3.920	1.337	0.039	0.208	−2.264
↓4-(dimethylamino)butanoate	3.890	1.215	0.000	0.241	−2.056

**Table 4:** Results of metabolite statistical analysis comparing the model control group to the antioxidant nutrient group (top 10 rows).

Compound Name	VIP	*p*-Value	Q-Value	FOLD CHANGE	LOG_FOLDCHANGE
↓Ethanolamine	0.022	0.414	0.913	0.493	−1.020
↑Glycine	0.724	0.700	0.933	1.140	0.189
↓β-Alanine	0.150	0.783	0.982	0.895	−0.160
↓L-Alanine	0.637	0.916	0.982	0.959	−0.061
↓D-Alanine	0.797	0.446	0.913	0.557	−0.844
↓L-2-Aminobutyric acid	1.655	0.185	0.913	0.513	−0.963
↓2-Aminoisobutyric acid	0.122	0.541	0.933	0.684	−0.549
↓(S)-beta-Aminoisobutyric acid	1.504	0.347	0.913	0.115	−3.116
↑γ-Aminobutyric acid	0.788	0.640	0.933	1.190	0.251
↓L-Serine	0.436	0.826	0.982	0.889	−0.170

**Table 5:** Results of metabolite statistical analysis comparing the model control group to the grape exosome group (top 10 rows).

Compound Name	VIP	*p*-Value	Q-Value	FOLD CHANGE	LOG_FOLDCHANGE
↓Ethanolamine	0.147	0.644	0.998	0.712	−0.491
↑Glycine	0.675	0.710	0.998	1.155	0.208
↓β-Alanine	0.008	0.945	0.998	0.968	−0.047
↓L-Alanine	0.548	0.698	0.998	0.846	−0.242
↑D-Alanine	0.399	0.910	0.998	1.088	0.122
↑L-2-Aminobutyric acid	0.241	0.588	0.998	1.345	0.427
↓2-Aminoisobutyric acid	0.216	0.878	0.998	0.911	−0.135
↓(S)-beta-Aminoisobutyric acid	1.218	0.341	0.998	0.104	−3.269
↓γ-Aminobutyric acid	0.544	0.937	0.998	0.960	−0.059
↓L-Serine	0.314	0.872	0.998	0.913	−0.132

**Table 6:** Summary of the correspondence between differential metabolites and pathways.

Metabolomics Type	Key Differential Metabolites	Corresponding Altered Metabolic Pathways
Untargeted Metabolomics	Riboflavin (upregulated)	Metabolic Pathway
	Pentadecanoic acid (downregulated)	Metabolic Pathway
	Isovaleric acid (downregulated)	Phenylalanine metabolism, Tryptophan metabolism
Targeted Metabolomics	Glycine (upregulated)	D-glutamine and D—glutamate metabolism, Biosynthesis of phenylalanine/tyrosine/tryptophan
	D-alanine (upregulated)	D-glutamine and D—glutamate metabolism
	L-2-aminobutyric acid (upregulated)	Biosynthesis of valine, leucine and isoleucine
	γ-aminobutyric acid (GABA) (downregulated)	D-glutamine and D—glutamate metabolism

**Table 7:** PPI network scores of the top 10 genes.

Gene	Betweenness	Gene	Betweenness
ITCH	3837.60	ZNF148	1206.00
PBXIP1	2546.00	KIF3A	1130.00
RAB8A	2076.00	SLC6A1	1102.00
KDR	1939.67	CBL	994.13
ETV6	1475.13	ESR1	858.00

## Data Availability

The original contributions presented in this study are included in the article. Further inquiries can be directed to the corresponding author.
